# The SLC25A1-p53 mutant crosstalk

**DOI:** 10.18632/aging.100785

**Published:** 2015-08-16

**Authors:** Chris Albanese, Maria Laura Avantaggiati

**Affiliations:** Department of Oncology, Georgetown University Medical Center, Lombardi Comprehensive Cancer Center, Washington, DC, USA

The presence of p53 mutations in human cancers, which occurs with a frequency higher than 50%, is a negative prognostic factor predictive of relapse and of resistance to chemo- and radio-therapy, thus rendering these types of tumors a still unsolved therapeutic challenge. While various compounds have become available for the treatment of tumors expressing other types of “driver” oncogenic mutations, such as EGFR, ALK or B-Raf, p53 mutant proteins are still “undraggable”. Thus, the identification of the pathways employed by mutant p53 (mutp53) to promote tumor progression has fundamental therapeutic implications. Many previous studies have focused on the interference of mutp53 with cell cycle progression, apoptosis and senescence. In the case of wild-type p53 it is now clear, however, that modulation of cellular metabolism fundamentally contributes to its tumor suppressive activity independently of its ability to regulate other cellular programs important for oncogenesis, specifically apoptosis and senescence [2]. How mutp53 regulates oncogenic metabolic circuits is still a relatively understudied area of investigation.

We have shown that various p53 mutants promote transcriptional activation of the mitochondrial citrate carrier SLC25A1, (CTP/CIC), which regulates the efflux of citrate from the mitochondria to the cytoplasm [3]. Treatment of cancer cell lines harboring p53 mutations with chemical inhibitors of SLC25A1, used as single agents, reduces tumor growth in vivo and enhances chemosensitivity to cisplatin. Further, high SLC25A1 expression levels confer resistance to platinum agents and powerfully predict the poorest survival outcome in lung cancer. Thus, our studies open new therapeutic opportunities and identity SLC25A1 as a potentially druggable target able to overcome, at least in part, the oncogenic activity of a subset of p53 mutants.

The unique activities of SLC25A1 render its regulation by mutp53 intriguing, while raising a number of interesting questions. We have proposed that SLC25A1 is one of only a handful of gene targets identified thus far, associated with mutp53 GOF activity and specifically involved in regulation of cellular metabolism. A previous study showed that mutp53 recruits the SREBP-1 transcription factor to enact a metabolic transcription program that promotes sterol and lipid biosynthesis [4]. Given that cytoplasmic citrate is the predominant source for lipid synthesis, it is likely that SLC25A1 is involved in this activity of mutp53. A second fundamental function of SLC25A1 surprisingly consists in overcoming the glycolytic addiction of tumors (the Warburg effect), while promoting mitochondrial activity and oxidative phosphorylation, through which SLC25A1 enacts adaptation in response to glucose starvation or mitochondrial respiration injury [5]. These two forms of stress ensue in hypovascular tumor regions and pose an important obstacle to the expansion of cancer cells. We have therefore proposed that SLC25A1 is necessary for metabolic plasticity and mitochondrial homeostasis, enabling adaptation and survival during nutritional and oxidative stress signals that progressing tumors face as their mass increases and the vasculature becomes inadequate to supply nutrients. Accordingly, SLC25A1 inhibition leads to a catastrophic collapse of the mitochondrial metabolism and to tumor inhibition. Although this SLC25A1 activity may at first glance appear paradoxical-given that the Warburg effect is considered a hallmark of tumors-recent evidence has challenged the long-standing precept of the glycolytic requirement for tumor proliferation, and “a reversal of the Warburg effect” exists in the tumor-stroma cross-talk [e.g.,^5^].

How do p53 mutants regulate glucose and mitochondrial metabolism? The observation that SLC25A1 reverts the Warburg effect and promotes mitochondrial respiration, raises the question of whether mutp53 plays a similar effect and thus also, in part, shares the activities of its native wild-type p53 counterpart. Accordingly, in patients affected by Li–Fraumeni syndrome harboring mutations of the p53 gene, mitochondrial respiration is increased in the skeletal muscle associated with high levels of mitochondrial respiratory complexes [6]. Even more intriguingly, mice harboring the p53R172H mutation (equivalent to the human codon R175H) display enhanced endurance during exercise and wild-type p53 was shown previously to promote a similar phenotype [2]. These observations intriguingly highlight that wild-type and mutant p53 may share pro-survival activities that enable adaptation to metabolic stress, although to what extent SLC25A1 is involved in these effects is still unclear. On the other hand, a second study has shown that tumor-derived p53 mutants stimulate the Warburg effect in cancer cells in conditions in which glucose is abundant, and genetic inhibition of glucose transporters compromises p53-mutant driven oncogenesis [7].

**Figure 1:**
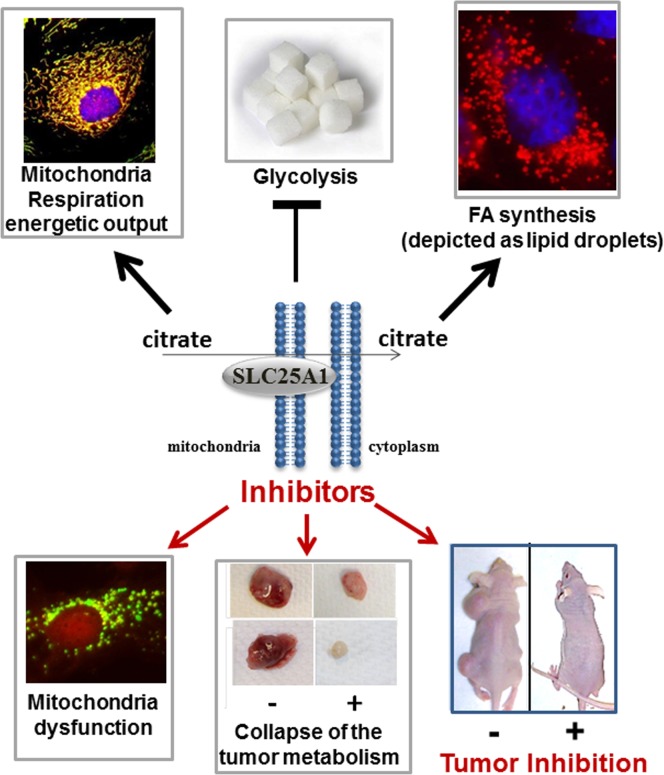
Summary of the activities of SLC25A1. Some of the images shown were previously published by our group [3,5].

These studies suggest that regulation of cellular metabolism by mutp53 may be complex, and depend strongly upon the type of tissue, the “geographical” metabolic heterogeneity of tumor cells as well as the stage of tumor initiation/progression. Finally, we have reported additional key activities of SLC25A1 that are relevant to oncogenesis, consisting of regulation of mitochondrial autophagy, inflammation as well as of the intracellular pools of Acetyl-Coenzyme A, the donor for all trans-acetylation reactions [5,8]. Therefore, SLC25A1 likely provides a nodal point at the cross-road of multiple signal pathways altered by mutant p53.
